# Prospective Evaluation of Peripheral Arterial Intervention Using Air Plethysmography

**DOI:** 10.3390/medicina62091795

**Published:** 2026-09-18

**Authors:** Anastasios G. Potouridis, Dimitrios A. Chatzelas, Apostolos G. Pitoulias, Maria D. Velikoudi, Georgios A. Pitoulias

**Affiliations:** 1 2nd Department of Surgery—Division of Vascular Surgery, “G. Gennimatas” General Hospital, Faculty of Medicine, Aristotle University of Thessaloniki, 54635 Thessaloniki, Greece; dchatzelas@outlook.com (D.A.C.); mariaveli95@gmail.com (M.D.V.); pitoulias@yahoo.com (G.A.P.); 2 Department of Vascular and Endovascular Surgery, St Bernward Hospital, 31134 Hildesheim, Germany; appitoulias@yahoo.com

**Keywords:** air plethysmography, pulse volume recording, peripheral arterial disease, peripheral arterial intervention, postoperative surveillance

## Abstract

*Background and Objectives:* Air plethysmography (APG), also referred to as pulse volume recording, provides a physiological assessment of lower-extremity perfusion, but its role in contemporary surveillance after peripheral arterial revascularization remains uncertain. This prospective study aimed to evaluate the ability of APG to monitor hemodynamic recovery and predict procedural failure after open and endovascular interventions. *Materials and Methods:* Consecutive patients undergoing infrainguinal revascularization for symptomatic peripheral arterial disease were prospectively enrolled and followed for 24 months. Standardized clinical assessment, ankle–brachial index, Duplex ultrasonography, and APG were performed preoperatively and at 1, 6, 12, and 24 months. Longitudinal hemodynamic changes, diagnostic accuracy for restenosis, patency, and predictors of procedural failure were analyzed. *Results:* A total of 120 patients were included, of whom 108 (90.0%) completed a 24-month follow-up. Mean pulse volume amplitude increased from 5.8 ± 2.3 mm preoperatively to 14.6 ± 4.1 mm at one month (*p* < 0.001) and remained significantly improved throughout follow-up. Systolic upstroke time decreased from 192 ± 31 to 128 ± 24 ms (*p* < 0.001), while waveform morphology also improved substantially. Longitudinal analysis demonstrated a significant effect of time (*p* < 0.001) and group-by-time interaction (*p* = 0.031). Among secondary outcomes, APG deterioration preceded fulfillment of the predefined Duplex criteria for significant restenosis in 26 of 34 failures, with a mean observed interval of 3.8 ± 1.2 months. A ≥25% reduction in pulse volume amplitude showed good discrimination for restenosis, while APG deterioration was independently associated with procedural failure (HR = 2.80, 95% CI 1.65–5.37; *p* < 0.001). *Conclusions:* APG is a reproducible, non-invasive surveillance adjunct that objectively reflects hemodynamic recovery and may identify physiological deterioration before fulfillment of predefined Duplex criteria for significant restenosis. These findings support further evaluation of APG as a complementary physiological modality within structured surveillance following lower-extremity revascularization.

## 1. Introduction

Peripheral arterial disease (PAD) is a common manifestation of systemic atherosclerosis, and remains a major cause of cardiovascular morbidity, functional impairment, and limb loss worldwide [1]. Contemporary management includes both open surgical and endovascular revascularization, aiming to restore limb perfusion, relieve symptoms, improve quality of life, and prevent major amputation [1,2,3]. Despite continuous advances in revascularization techniques, restenosis, intimal hyperplasia, and graft or target-lesion failure remain major determinants of long-term durability and important causes of recurrent ischemia, emphasizing the need for reliable postoperative surveillance [2,3,4,5].

Current follow-up protocols rely primarily on clinical examination, ankle-brachial index (ABI), and Duplex ultrasonography [1,2,5]. Duplex imaging is considered the reference standard for surveillance because it can accurately identify graft stenosis and restenosis after intervention [5]. However, it is operator-dependent, relatively time-consuming, and evaluates individual arterial segments, rather than the global physiological recovery of limb perfusion [4,5]. ABI is inexpensive and widely available, but may be unreliable in patients with diabetes mellitus, chronic kidney disease (CKD), or severe arterial calcification because of vessel incompressibility [4,6].

Air plethysmography (APG), also referred to as pulse volume recording (PVR), is a non-invasive technique that measures pulsatile limb volume changes generated during the cardiac cycle [7]. By recording volumetric rather than pressure changes, APG provides functional information regarding arterial inflow, vascular compliance, pulse wave transmission, and peripheral resistance [7,8,9,10]. Unlike ABI, its measurements are largely unaffected by medial arterial calcification, making it an attractive complementary tool for hemodynamic assessment [6,7,8,9,10]. Historically, APG was widely used for the diagnosis of PAD and the evaluation of arterial reconstruction, where restoration of normal pulse volume waveforms was shown to correlate with successful revascularization and durable graft patency [11,12,13,14,15,16]. However, its use gradually declined following the widespread adoption of Duplex ultrasonography, despite the fact that both techniques evaluate different aspects of vascular physiology [4,8,10]. For consistency, the term APG is used throughout the present manuscript to refer to the examination technique, whereas PVR refers specifically to the resulting arterial waveform.

Although Duplex ultrasonography has largely replaced APG in routine clinical practice, renewed interest in physiological assessment of limb perfusion has highlighted the potential advantages of volumetric techniques [4,17,18]. Existing evidence suggests that deterioration of pulse volume waveforms may precede clinical recurrence and imaging-confirmed restenosis, but most available studies were performed several decades ago, were retrospective in design, or included relatively small patient cohorts [13,14,15,16,18]. Moreover, there is a paucity of contemporary prospective studies evaluating APG using standardized acquisition protocols and comparing its performance with current postoperative surveillance, after both open and endovascular lower-extremity revascularization [5,6,18].

The present prospective study was designed to evaluate the role of APG in the postoperative assessment of peripheral arterial interventions during a structured 24-month surveillance program. The primary objective was to investigate longitudinal changes in APG-derived hemodynamic parameters following successful revascularization. Secondary objectives were to compare hemodynamic recovery after open and endovascular procedures, determine the relationship between APG findings and conventional surveillance modalities, and evaluate the association of APG deterioration with subsequent restenosis and procedural failure.

## 2. Materials and Methods

### 2.1. Study Design and Patient Population

This prospective observational study was conducted in a single tertiary university vascular surgery department between 1 January 2022 and 31 December 2022. Consecutive patients undergoing elective or urgent lower-extremity revascularization for symptomatic PAD were prospectively enrolled during that period and followed prospectively until 31 December 2024. The study protocol was approved by the institutional ethics committee, and written informed consent was obtained from all participants before enrolment. The study was conducted in accordance with the principles of the Declaration of Helsinki and is reported in accordance with the STROBE recommendations for observational cohort studies [19,20].

Patients were eligible if they were at least 18 years of age and had symptomatic infrainguinal lower-extremity PAD requiring revascularization for lifestyle-limiting intermittent claudication (IC; Rutherford stages 2–3) or chronic limb-threatening ischemia (CLTI; Rutherford stages 4–6) [21]. The decision to perform open or endovascular revascularization was made by the institutional vascular team according to lesion location and anatomical complexity, patient comorbidities and operative risk, life expectancy, availability of suitable autologous conduit, previous ipsilateral interventions, and current international guidelines [1].

Patients with acute limb ischemia, non-atherosclerotic arterial disease, active systemic infection, severe congestive heart failure, inability to co-operate during plethysmographic examination, or expected survival shorter than one year were excluded. Limbs with extensive ulceration preventing proper cuff placement were also excluded from APG assessment.

### 2.2. Study Groups

Patients were allocated into two predefined treatment groups. The open revascularization group included patients undergoing common femoral endarterectomy with patch angioplasty, femoropopliteal bypass, femorodistal bypass, or hybrid open procedures in which the principal component of treatment consisted of surgical reconstruction. The endovascular group included patients treated with balloon angioplasty alone or in combination with bare-metal stents, covered stents, atherectomy, or combinations of these techniques, according to lesion characteristics and operator preference. Hybrid procedures were classified according to the predominant mode of revascularization.

Technical success was defined as successful completion of the planned procedure with restoration of inline arterial flow to the distal target vessel. For endovascular procedures, technical success required residual angiographic stenosis < 30% on completion angiography without flow-limiting dissection or other technical complication. For open reconstruction, technical success was defined as a patent reconstruction with satisfactory intraoperative flow and no technical defect requiring immediate revision. For hybrid procedures, both the open and endovascular components were required to fulfill their respective technical success criteria [1].

### 2.3. Conventional Vascular Assessment

All patients underwent standardized clinical assessment, ABI measurement, and Duplex ultrasonography at every follow-up visit. ABI was measured using a handheld continuous-wave Doppler probe after at least ten minutes of supine rest. The higher ankle systolic pressure was divided by the higher brachial systolic pressure.

Duplex ultrasonography was performed by certified vascular surgeons using a high-resolution color Duplex system. Sonographers were blinded to the APG findings. Peak systolic velocity (PSV) was calculated across all treated arterial segments. Restenosis was defined as a PSV ratio ≥ 2.5, corresponding to at least 50% luminal narrowing, whereas severe restenosis was defined by a ratio ≥ 3.5 or diameter reduction ≥ 70%, according to established vascular laboratory criteria [22,23]. When clinically indicated, computed tomography angiography (CTA) or digital subtraction angiography was performed for confirmation before reintervention.

### 2.4. Air Plethysmography Protocol

All plethysmographic examinations were performed in the vascular laboratory by two experienced vascular surgeons, who were unaware of the Duplex ultrasonography findings. Patients were instructed to avoid smoking, caffeine-containing beverages, and strenuous physical activity for at least four hours before examination. All measurements were obtained in a quiet, temperature-controlled laboratory maintained between 22 and 24 °C to minimize sympathetic vasomotor influences. Following a resting period of ten minutes in the supine position, appropriately sized pneumatic cuffs were applied circumferentially around the thigh, calf, and transmetatarsal region [8,9,10].

APG recordings were obtained using an APG-1000™ pneumatic plethysmographic system (ACI Medical, Sun Valley, CA, USA), with cuff inflation maintained at approximately 60 mmHg, allowing transmission of pulsatile arterial volume changes while avoiding significant vascular compression [9]. Appropriately sized pneumatic cuffs were used according to the manufacturer’s recommendations, and the system was calibrated according to the manufacturer’s standard procedure before examination. Three consecutive recordings were obtained at the thigh, calf, and transmetatarsal levels, and the average values were used for subsequent analysis. Calf recordings were used for the principal quantitative longitudinal analysis. Pulse volume amplitude was measured directly as the vertical peak-to-trough excursion of the recorded waveform, and expressed in millimeters; no proprietary derived amplitude index was used. Whenever excessive movement or recording artifacts occurred, acquisition was repeated until technically satisfactory waveforms were obtained.

**Figure 1:**
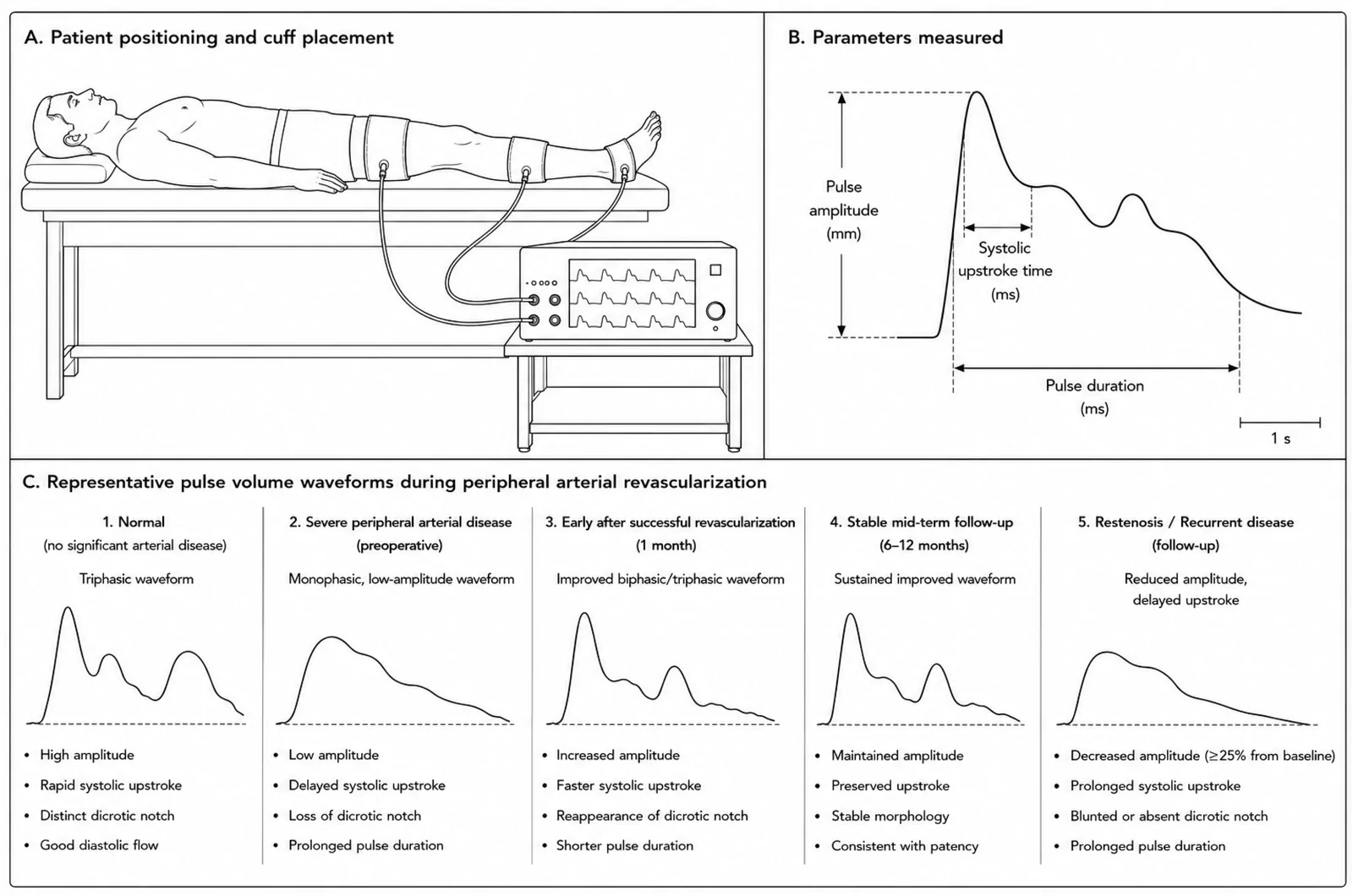
Methodology of air plethysmography and representative pulse volume recordings during peripheral arterial revascularization: (**A**) Patient positioning and standardized placement of pneumatic cuffs, (**B**) Quantitative waveform parameters measured during analysis, (**C**) Representative pulse volume waveforms demonstrating normal arterial perfusion, severe peripheral arterial disease, early postoperative improvement following successful revascularization, stable mid-term patency, and waveform deterioration associated with restenosis.

Both qualitative waveform morphology and quantitative waveform characteristics were analyzed. Waveform morphology was assessed as a qualitative component of the APG examination and of the composite deterioration criterion, whereas pulse volume amplitude represented the principal quantitative parameter for longitudinal and diagnostic-performance analyses. Qualitative assessment classified waveforms as triphasic, biphasic, monophasic, or severely dampened according to accepted vascular laboratory criteria. Quantitative analysis included pulse volume amplitude, systolic upstroke time, pulse wave duration, and percentage change in waveform amplitude compared with the pre-operative examination [7,8,9,10,18]. Segmental PVR was used to identify the anatomical level of hemodynamic impairment and to assess changes following revascularization. The standardized APG examination protocol and the representative pulse volume recordings analyzed during the study are illustrated in Figure 1.

The one-month postoperative examination was selected a priori as the reference for subsequent longitudinal comparisons, rather than the immediate postoperative assessment, to allow resolution of acute perioperative influences on plethysmographic measurements, including postoperative edema, inflammation, alterations in vascular tone, and early hemodynamic adaptation following revascularization.

For clinical surveillance purposes, APG deterioration was prospectively defined as a composite trigger comprising any of the following: a reduction in pulse volume amplitude of at least 25% compared with the one-month postoperative examination, deterioration in waveform morphology from triphasic or biphasic to monophasic, or prolongation of systolic upstroke exceeding 20% in association with clinical suspicion of restenosis. Patients fulfilling any of these criteria underwent expedited Duplex ultrasonography irrespective of the scheduled surveillance visit.

Interobserver reproducibility was evaluated in a subset of 20 patients selected by simple random sampling from the overall study cohort. APG examinations were independently analyzed by both vascular surgeons, who were blinded to each other’s measurements. Agreement for pulse volume amplitude and systolic upstroke time was assessed using a two-way mixed-effects intraclass correlation coefficient (ICC),for absolute agreement based on single measurements, while agreement for qualitative waveform classification was evaluated using weighted Cohen’s κ coefficient.

### 2.5. Follow-Up Protocol

Patients were evaluated before intervention and subsequently at one month, six months, twelve months, and twenty-four months. Each visit included clinical examination, wound assessment when applicable, ABI measurement, APG examination, and Duplex ultrasonography. If APG demonstrated significant deterioration despite normal Duplex findings, repeat Duplex ultrasonography was scheduled within six weeks. Conversely, patients with Duplex-confirmed restenosis underwent management according to standard institutional practice, including intensified medical therapy, endovascular revision, surgical revision, or continued surveillance depending on lesion severity and symptoms.

### 2.6. Outcome Measures

The primary outcome of the study was the longitudinal change in arterial pulse volume waveform characteristics following revascularization. Secondary outcomes included primary patency, assisted primary patency, secondary patency, freedom from target lesion revascularization (TLR), overall survival, and the diagnostic performance of APG for identifying restenosis. Primary patency was defined as uninterrupted patency of the treated arterial segment or bypass graft without significant restenosis, occlusion, or reintervention to maintain or restore patency; accordingly, Duplex-confirmed significant restenosis according to the predefined criteria constituted loss of primary patency. Assisted primary patency was defined as maintained patency following an intervention performed to prevent impending occlusion of a still-patent treated segment or bypass graft. Secondary patency was defined as restoration and subsequent maintenance of patency following occlusion. TLR was defined as any repeat endovascular or surgical intervention performed for clinically significant restenosis or occlusion involving the originally treated arterial segment; for surgical reconstructions, this included the bypass graft, whereas for endovascular procedures it included the previously treated native arterial segment. For the purposes of the Cox proportional hazards analysis, procedural failure was defined as loss of primary patency due to Duplex-confirmed significant restenosis according to the predefined criteria, occlusion of the treated arterial segment or bypass graft, or reintervention performed to maintain or restore patency.

An additional predefined endpoint was the interval between the first abnormal APG examination and subsequent fulfillment of the predefined Duplex criteria for significant restenosis or confirmation by cross-sectional imaging. This interval was calculated from the date of the first APG examination meeting the predefined deterioration criteria to the date of the first subsequent imaging examination fulfilling the predefined restenosis criteria, irrespective of whether the latter examination was performed as part of the scheduled surveillance protocol or as an unscheduled APG-triggered assessment. Patients lost to follow-up were censored at the last available visit. Missing longitudinal measurements were handled within the mixed-effects model without imputation.

### 2.7. Statistical Analysis

Continuous variables were tested for normality using the Shapiro–Wilk test and are presented as mean ± standard deviation or median with interquartile range, as appropriate. Categorical variables are presented as frequencies and percentages. Baseline characteristics between treatment groups were compared using the independent-samples Student’s t-test or the Mann–Whitney U test for continuous variables and the χ^2^ test or Fisher’s exact test for categorical variables. Longitudinal changes in APG parameters, ABI, and Duplex measurements were analyzed using linear mixed-effects models with treatment group, follow-up interval, and group-by-time interaction included as fixed effects. A patient-specific random intercept was included to account for within-patient correlation arising from repeated measurements over time. Follow-up interval was treated as a categorical variable. Post hoc comparisons were adjusted using the Bonferroni correction. Correlations between APG parameters and ABI or Duplex-derived PSV ratios were evaluated using Pearson’s or Spearman’s correlation coefficients, depending on data distribution.

Diagnostic accuracy analysis was performed separately for percentage change in pulse volume amplitude. Receiver operating characteristic (ROC) analysis evaluated percentage reduction in pulse volume amplitude as a continuous variable in relation to subsequent fulfillment of the predefined Duplex criteria for significant restenosis. Diagnostic performance was additionally assessed at the prespecified ≥25% amplitude-reduction threshold. Diagnostic accuracy analyses were performed on a per-patient basis, with each patient contributing once to the analysis; repeated APG examinations were not treated as independent observations. For patients who developed significant restenosis, only APG changes occurring before or at the examination at which the predefined Duplex criteria were first fulfilled were considered for diagnostic classification. The diagnostic endpoint was assessed over the predefined 24-month surveillance period.

Primary, assisted primary, and secondary patency, as well as freedom from TLR, were estimated using the Kaplan–Meier method and compared with the log-rank test. A prespecified 6-month landmark analysis was performed to evaluate subsequent primary patency according to APG status. Only patients who were alive, remained under surveillance, and were free from primary-patency failure at six months were included in the landmark cohort; patients who died, were lost to follow-up, or experienced primary-patency failure before the landmark were excluded from this analysis. APG status at the landmark was classified as stable or deteriorated according to whether the predefined APG deterioration criteria had been fulfilled by the 6-month examination. Follow-up for the landmark analysis commenced at six months after the index procedure.

Associations with procedural failure, as defined above, were evaluated using Cox proportional hazards regression. Candidate variables for multivariable analysis were selected on the basis of univariable association (*p* < 0.10) and clinical relevance to revascularization failure. Postoperative APG deterioration was modeled as a time-dependent covariate, with patients considered unexposed until the first examination fulfilling the predefined APG deterioration criteria and exposed thereafter. Continuous covariates were assessed for linearity before inclusion in the model. There were no missing data for variables included in the Cox regression analysis; therefore, no imputation was required. The proportional hazards assumption was assessed individually for each covariate by testing its interaction with time, with no significant violations identified. Hazard ratios are reported with corresponding 95% confidence intervals. Given the limited number of outcome events relative to the number of covariates, the multivariable analysis was considered exploratory. A two-sided *p*-value < 0.050 was considered statistically significant. Statistical analyses were performed using IBM SPSS Statistics version 30.0 (IBM Corp., Armonk, NY, USA).

## 3. Results

### 3.1. Patient Population

Between 1 January 2022 and 31 December 2022, 136 consecutive patients fulfilled the inclusion criteria. Nine declined participation and seven were excluded because complete postoperative APG examinations could not be obtained. Thus, the final study population consisted of 120 patients, corresponding to 120 treated limbs, who completed the baseline evaluation and underwent the scheduled intervention. During the 24-month follow-up, five patients (4.2%) died from causes unrelated to the index procedure and seven (5.8%) were lost to follow-up. Consequently, 108 patients (90.0%) completed the scheduled two-year follow-up.

Sixty patients (50.0%) underwent open surgical reconstruction and 60 underwent endovascular revascularization. Baseline demographic, clinical, and lesion characteristics are summarized in Table 1 and were well balanced between treatment groups. The mean age of the study population was 69.1 ± 8.7 years, and 84 patients (70.0%) were men. Diabetes mellitus was present in 55 patients (45.8%), hypertension in 95 (79.2%), dyslipidemia in 87 (72.5%), coronary artery disease in 42 (35.0%), CKD in 23 (19.2%), and 76 patients (63.3%) were current or former smokers. Forty-seven patients (39.2%) presented with IC (Rutherford stages 2–3), whereas seventy-three (60.8%) had CLTI (Rutherford stages 4–6). Mean preoperative ABI was 0.54 ± 0.16, with no statistically significant difference between treatment groups (*p* = 0.42).

### 3.2. Procedural Characteristics

Among the 60 patients undergoing open reconstruction, 34 (56.7%) underwent femoropopliteal bypass, 15 (25.0%) femorodistal bypass, 8 (13.3%) common femoral endarterectomy with patch angioplasty, and 3 (5.0%) hybrid reconstructions consisting of common femoral endarterectomy combined with iliac stenting. Of the 49 bypass procedures, autologous great saphenous vein was used in 19 (38.8%), whereas prosthetic grafts were implanted in the remaining 30 (61.2%). The endovascular cohort comprised 31 (51.7%) balloon angioplasties with plain balloons, 4 (6.7%) balloon angioplasties with drug-coated balloons, 18 (30.0%) primary self-expanding stent implantations, and 7 (11.7%) atherectomy-assisted procedures. The superficial femoral artery represented the most frequently treated arterial segment (58.3%), followed by the popliteal artery (26.7%) and infrapopliteal arteries (15.0%). The mean treated lesion length was 14.2 ± 6.8 cm overall.

Technical success was achieved in 58 patients (96.7%) in the open group and 57 (95.0%) in the endovascular group (*p* = 0.64). Median postoperative hospital stay was shorter in the endovascular group: 2 (1–3) vs. 5 (4–7) days, *p* < 0.001. Early procedural outcomes were favorable in both groups. Thirty-day mortality was 1.7% in each group. Major adverse cardiovascular events occurred in 2 patients undergoing open reconstruction and 1 undergoing endovascular treatment. Early graft or target-lesion thrombosis developed in 2 patients (3.3%) after open surgery and 3 patients (5.0%) after endovascular intervention (*p* = 0.65). Surgical-site infection occurred in 3 patients (5.0%) following open reconstruction, whereas 2 access-site complications (3.3%) were observed after endovascular treatment; neither difference reached statistical significance. No significant differences were observed in the overall rate of major perioperative complications between treatment groups (*p* = 0.73).

### 3.3. Perioperative APG Findings

Complete clinical and hemodynamic follow-up was available for all surviving patients at the 1-month visit, establishing the postoperative reference examination used for subsequent longitudinal analyses. Successful revascularization resulted in marked early postoperative improvement in all APG parameters. Mean pulse volume amplitude increased from 5.8 ± 2.3 mm preoperatively to 14.6 ± 4.1 mm one month after intervention, corresponding to a mean increase of 8.8 mm (95% CI 8.0–9.6 mm), or approximately 152% above baseline (*p* < 0.001). Improvement remained evident throughout follow-up, although a gradual decline was observed after the first postoperative year. Similarly, mean systolic upstroke time decreased significantly from 192 ± 31 ms before intervention to 128 ± 24 ms at one month (mean difference −64 ms, 95% CI −72 to −56 ms; *p* < 0.001), indicating restoration of rapid arterial inflow.

Waveform morphology improved substantially following successful revascularization. Before intervention, only 9 patients (7.5%) demonstrated triphasic waveforms, despite meeting the clinical, hemodynamic, and anatomical criteria for revascularization, whereas 71 (59.2%) had monophasic recordings. At one month, triphasic or biphasic waveforms were present in 92 patients (76.7%). Patients undergoing open reconstruction demonstrated greater early improvement in pulse volume amplitude than those treated endovascularly (9.8 ± 3.2 mm vs. 7.9 ± 3.0 mm, *p* = 0.004). Nevertheless, both treatment groups exhibited highly significant improvement compared with baseline, and the between-group difference progressively diminished during follow-up.

Interobserver reproducibility was excellent. The ICC was 0.94 (95% CI 0.88–0.97) for pulse volume amplitude and 0.91 (95% CI 0.84–0.96) for systolic upstroke time. Agreement for qualitative waveform classification was also excellent (weighted κ = 0.87).

### 3.4. Conventional Hemodynamic Assessment

ABI improved significantly following intervention, increasing from 0.54 ± 0.16 preoperatively to 0.88 ± 0.12 at one month (*p* < 0.001). During follow-up, ABI remained stable in most patients, declining only among those who subsequently developed restenosis. Pulse volume amplitude demonstrated a moderate-to-strong positive correlation with ABI (r = 0.63, *p* < 0.001). Similarly, a moderate inverse correlation was observed between pulse volume amplitude and Duplex-derived PSV ratio (r = −0.58, *p* < 0.001), indicating that lower APG amplitudes were associated with progressively increasing hemodynamic severity of restenosis.

### 3.5. Longitudinal Hemodynamic Changes

Repeated-measures mixed-effects analysis demonstrated a highly significant effect of time on pulse volume amplitude (*p* < 0.001), together with a significant group-by-time interaction (*p* = 0.031), indicating different patterns of hemodynamic recovery between treatment strategies. Although the overall treatment effect did not reach statistical significance (*p* = 0.114), patients undergoing open reconstruction maintained more stable pulse volume amplitudes throughout follow-up than those treated endovascularly.

**Figure 2:**
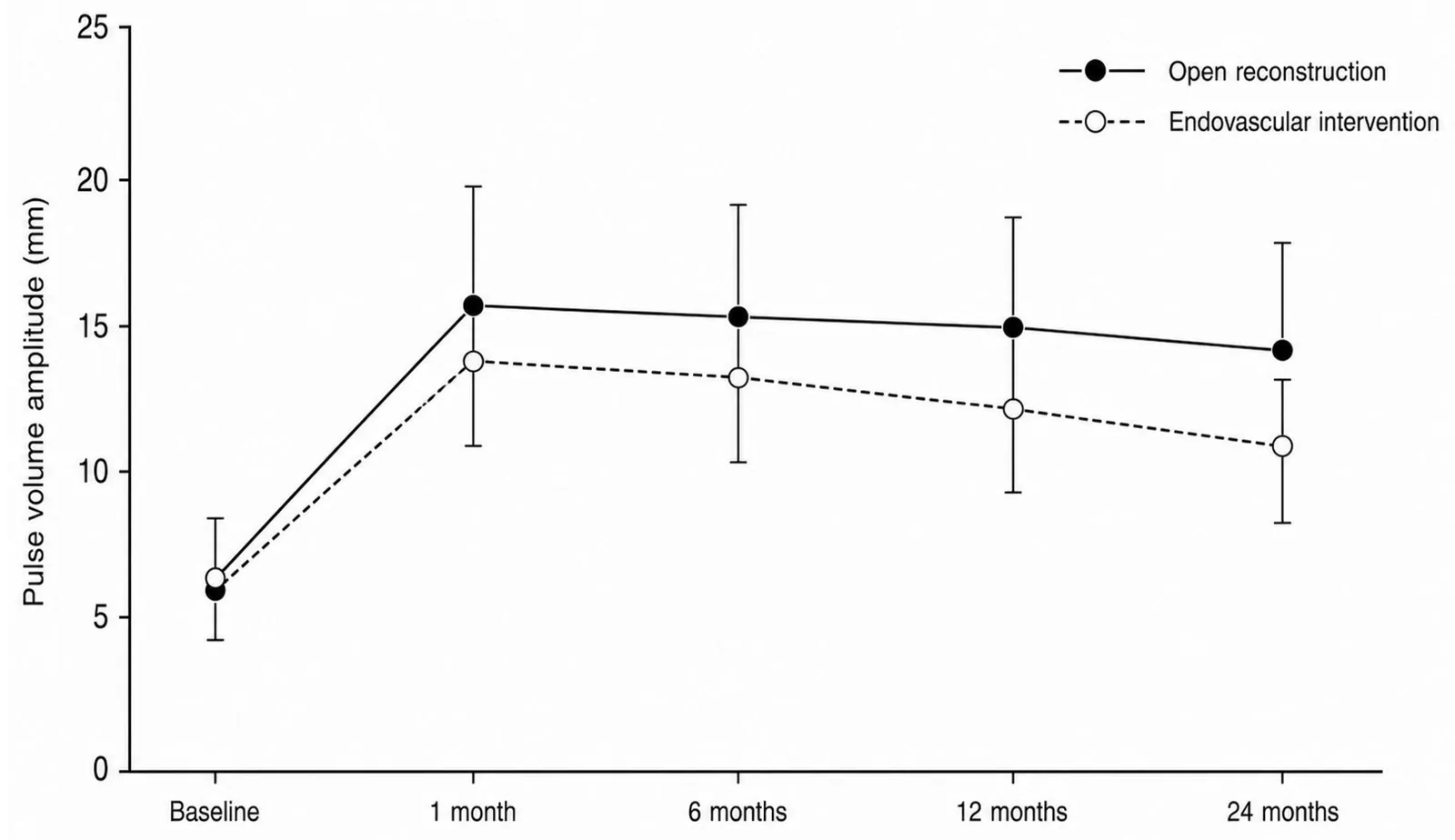
Longitudinal changes in pulse volume amplitude following peripheral arterial revascularization.

Pulse volume amplitude remained relatively unchanged after the first postoperative examination in the open group, declining by approximately 9% between 1 and 24 months. In contrast, the endovascular cohort exhibited a gradual reduction in pulse volume amplitude throughout follow-up, which became more pronounced after 12 months, resulting in an overall decline of 21% between the 1- and 24-month examinations (Figure 2). This longitudinal change was evaluated within the prespecified mixed-effects model, which accounted for repeated measurements within individual patients. Nevertheless, APG values remained significantly improved compared with baseline at every follow-up assessment in both treatment groups (all *p* < 0.001).

Unlike APG measurements, ABI demonstrated minimal variation after the 1-month postoperative examination, remaining relatively stable until restenosis developed. These findings suggest that APG may provide complementary physiological information in patients in whom ABI changes remain limited or difficult to interpret.

### 3.6. Detection of Restenosis

During follow-up, 34 restenosis or occlusion events occurred among the 108 patients with evaluable surveillance data (31.5%), including 14 events in the open-revascularization group and 20 in the endovascular group (25.9% vs. 37.0%, *p* = 0.18). APG deterioration was present in 28 of the 34 failure cases. In 26 patients (76.5% of all failures), APG abnormalities preceded fulfillment of the predefined Duplex criteria for restenosis, whereas APG and Duplex abnormalities were detected at the same visit in two patients. The earliest APG changes consisted of a progressive reduction in pulse volume amplitude, waveform flattening, and prolongation of systolic upstroke. Among the 26 patients in whom APG deterioration preceded fulfillment of the predefined Duplex criteria for restenosis, the mean observed interval between the first abnormal APG examination and subsequent fulfillment of the Duplex criteria was 3.8 ± 1.2 months. The corresponding intervals were similar after open and endovascular revascularization (4.0 ± 1.3 months vs. 3.7 ± 1.1 months, respectively; *p* = 0.47). Eighteen of the 26 patients with preceding APG abnormalities (69.2%) were asymptomatic when APG first became abnormal. Thus, APG deterioration was frequently documented before symptom recurrence or fulfillment of the predefined Duplex criteria for significant restenosis.

### 3.7. Diagnostic Performance of APG

**Figure 3:**
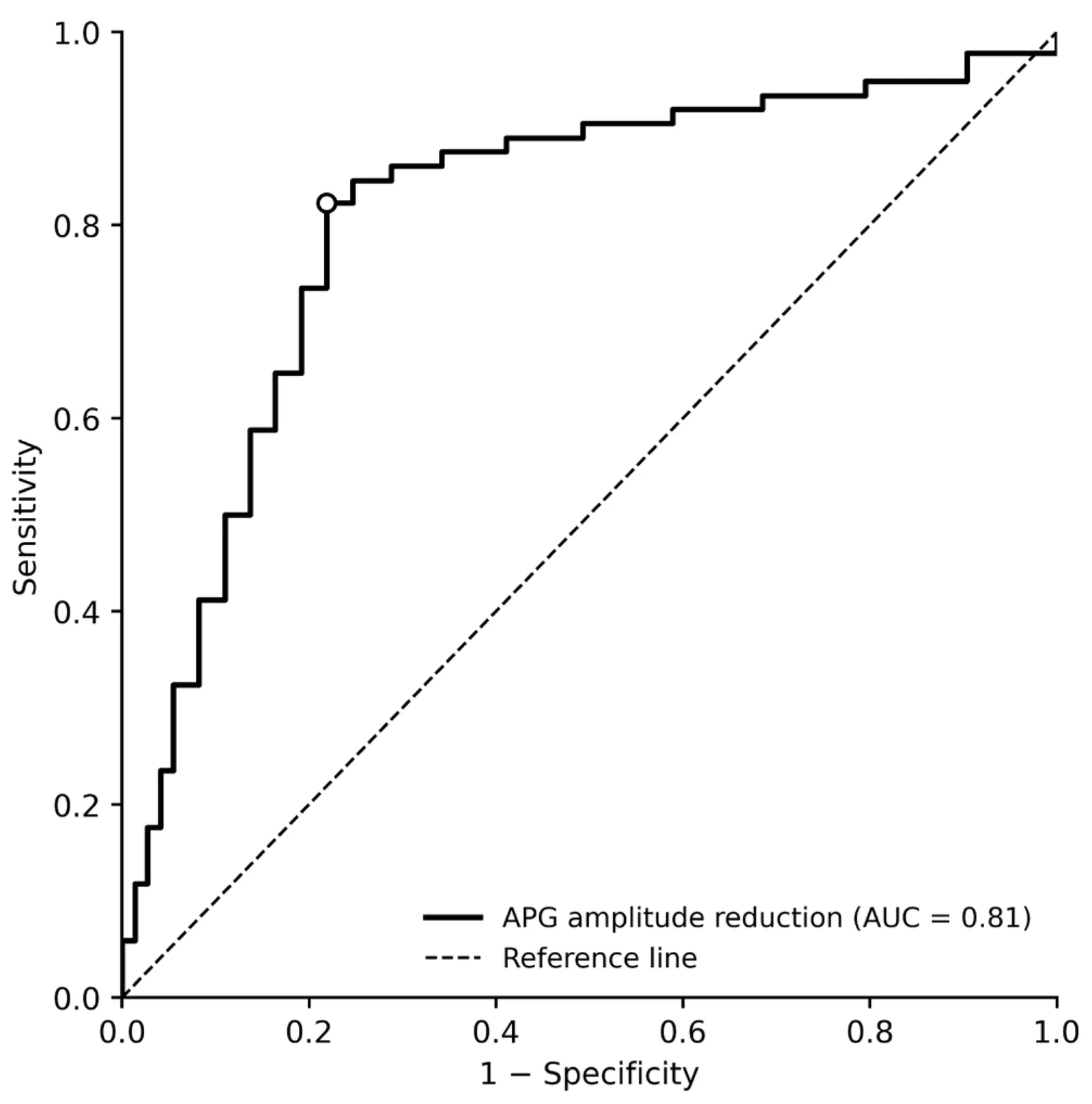
Receiver operating characteristic curve of percentage reduction in pulse volume amplitude for subsequent fulfillment of the predefined Duplex criteria for significant restenosis (Abbreviations: APG: air plethysmography; AUC: area under the curve).

ROC analysis of percentage change in pulse volume amplitude demonstrated good discrimination for subsequent fulfillment of the predefined Duplex criteria for significant restenosis, with an area under the curve of 0.81 (95% CI 0.72–0.90). At the prespecified ≥25% reduction threshold, sensitivity was 82.4%, specificity 78.4%, positive predictive value 63.6%, and negative predictive value 90.6% (Figure 3).

### 3.8. Clinical Outcomes

Among the 108 patients available for clinical assessment at 24 months, Rutherford category improved by at least one class in 93 (86.1%). Clinical improvement was observed in 49 of 54 patients (90.7%) following open reconstruction and in 44 of 54 (81.5%) after endovascular intervention, although the between-group difference was not statistically significant (*p* = 0.16). Among the 47 patients treated for intermittent claudication, 39 (83.0%) were free from lifestyle-limiting symptoms at the final assessment. Complete wound healing was achieved in 27 of the 33 patients presenting with tissue loss (81.8%), with a median time to healing of 5.8 months (3.9–8.4 months). Healing occurred in 14 of 16 patients (87.5%) in the open group and 13 of 17 (76.5%) in the endovascular group (*p* = 0.66). Five major amputations occurred during follow-up, all among patients with CLTI. Twenty-three patients required TLR, including 8 in the open group and 15 in the endovascular group. Reintervention consisted of endovascular revision in 14 patients, surgical revision in 6, and thrombectomy with adjunctive angioplasty in 3 patients.

### 3.9. Patency Analysis

During 24 months of surveillance, 14 primary-patency events occurred following open reconstruction and 20 following endovascular intervention. Patients who died without prior loss of patency or were lost to follow-up were censored at the date of death or last documented vascular assessment, respectively. Kaplan–Meier estimated primary patency at 24 months was 81.5% after open reconstruction and 68.9% after endovascular treatment (*p* = 0.041). Assisted primary patency was 90.4% and 80.2%, respectively (*p* = 0.083), while secondary patency was 94.2% and 88.0% (*p* = 0.204). Freedom from TLR at 24 months was 87.0% following open reconstruction and 74.1% following endovascular treatment (*p* = 0.048).

**Figure 4:**
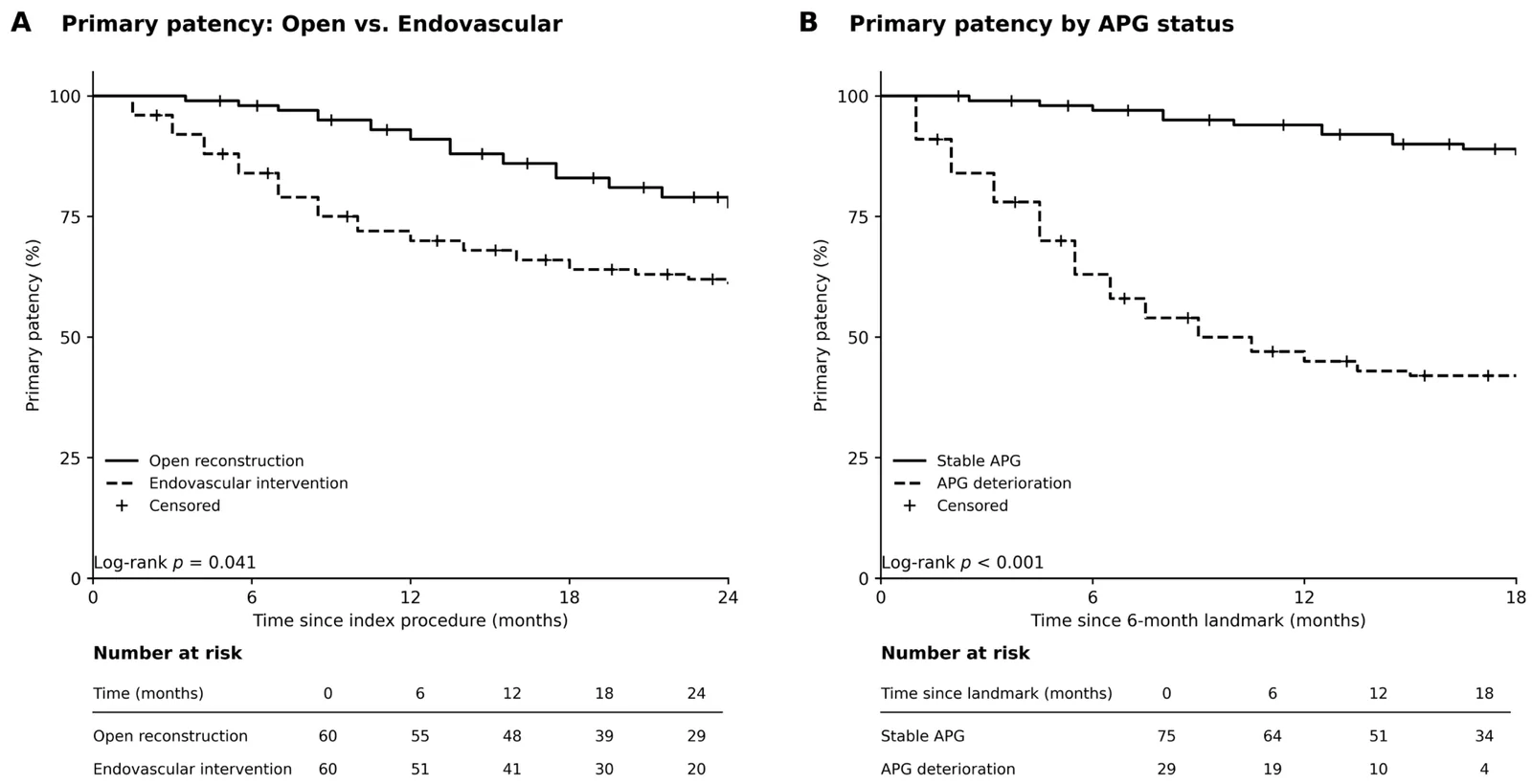
Kaplan–Meier estimates of primary patency following lower-extremity revascularization: (**A**) Primary patency after open surgical reconstruction and endovascular intervention from the time of the index procedure. (**B**) Prespecified 6-month landmark analysis comparing subsequent primary patency between patients with stable APG findings and those demonstrating APG deterioration among patients alive, under surveillance, and free from primary-patency failure at the landmark.

In the prespecified landmark analysis, patients demonstrating APG deterioration had substantially poorer subsequent patency than those with stable plethysmographic findings. Estimated 24-month primary patency was 42.7% in the APG-deterioration group compared with 88.3% among patients with stable APG recordings (*p* < 0.001). Assisted primary patency was 66.8% vs. 94.1% (*p* < 0.001), secondary patency was 78.4% vs. 95.0% (*p* = 0.012), and freedom from TLR was 59.7% vs. 91.6% (*p* < 0.001), respectively. Figure 4 presents the Kaplan–Meier estimates of primary patency according to treatment strategy (A) and the prespecified 6-month landmark analysis stratified by postoperative APG status (B).

### 3.10. Predictors of Procedural Failure

Thirty-four procedural-failure events were available for the Cox regression analysis. Variables demonstrating a univariable association at *p* < 0.10, together with clinically relevant covariates, were considered for multivariable analysis. Postoperative APG deterioration was incorporated as a time-dependent covariate. No significant violations of the proportional hazards assumption were identified for the individual covariates. Three variables remained independently associated with procedural failure. A reduction in pulse volume amplitude of at least 25% conferred the greatest risk of restenosis or occlusion (HR = 2.80, 95% CI 1.65–5.37; *p* < 0.001). Diabetes mellitus was associated with approximately a twofold increased risk (HR = 2.14, 95% CI 1.16–3.93; *p* = 0.015), while endovascular treatment remained independently associated with procedural failure (HR = 1.81, 95% CI 1.04–3.17; *p* = 0.037). Smoking, CKD, lesion length, and baseline ABI were not independently associated with procedural failure after multivariable adjustment. Table 2 summarizes the multivariable Cox proportional hazards regression analysis of predictors of procedural failure.

## 4. Discussion

The current prospective study demonstrates that APG provides clinically meaningful information following lower-extremity revascularization and may represent a valuable adjunct to contemporary postoperative surveillance. The principal findings are threefold. First, successful revascularization was associated with early postoperative and sustained improvement in plethysmographic waveforms and pulse volume amplitude, reflecting restoration of physiological limb perfusion. Second, deterioration of APG parameters frequently preceded fulfillment of the predefined Duplex criteria for significant restenosis, suggesting that physiological deterioration may become apparent before conventional imaging thresholds are reached. Finally, postoperative APG deterioration was independently associated with loss of primary patency during 24-month follow-up, suggesting that APG may have prognostic value beyond conventional non-invasive vascular assessment.

The objective assessment of revascularization has traditionally focused on anatomical success. Completion angiography, Duplex ultrasonography, and cross-sectional imaging can accurately demonstrate vessel patency and detect structural abnormalities [1,5]. However, these techniques evaluate morphology, rather than the integrated physiological consequences of revascularization [4,6,18]. Restoration of arterial continuity does not necessarily imply restoration of normal limb hemodynamics, particularly in patients with diffuse distal disease, impaired runoff, extensive arterial calcification, or microvascular dysfunction [4,17]. APG evaluates pulsatile volume change generated by each cardiac cycle, and therefore reflects the combined effects of arterial inflow, vascular compliance, pulse-wave transmission, peripheral resistance, and distal runoff [7,8,9,10,18,24]. Consequently, it provides information that complements, rather than duplicates, conventional imaging. This physiological perspective probably explains the close relationship observed in our study between APG improvement and subsequent clinical recovery.

The early postoperative improvement observed in pulse volume amplitude and waveform morphology is consistent with the classical vascular laboratory studies that established the physiological basis of arterial plethysmography [11]. Griffin et al. demonstrated that restoration of normal pulse volume recordings immediately following arterial reconstruction accurately reflected successful revascularization, and identified technical defects requiring intraoperative revision [13]. Similarly, Baird et al. reported substantial increases in waveform amplitude following femoropopliteal bypass, and showed that persistent waveform abnormalities frequently indicated inadequate reconstruction [14]. Although these investigations were performed several decades ago, before the widespread adoption of Duplex ultrasonography, they remain among the strongest demonstrations that plethysmographic waveforms closely reflect restoration of limb perfusion [12,16,18]. Our prospective findings extend these historical observations to contemporary vascular practice, incorporating both open surgical and endovascular procedures performed according to current treatment strategies.

Perhaps the most clinically important observation of the present study was that APG deterioration frequently preceded fulfillment of the predefined Duplex criteria for significant restenosis. In more than 75% of limbs that subsequently developed restenosis, waveform deterioration was documented before the Duplex threshold was reached, with a mean observed interval of nearly four months. Moreover, approximately 70% of these patients were asymptomatic when APG first became abnormal. These findings suggest that physiological deterioration may become apparent before symptom recurrence or before a lesion fulfills conventional Duplex criteria for significant restenosis. This temporal relationship may reflect the different information provided by the two modalities, with APG capturing integrated changes in limb hemodynamics while Duplex criteria classify restenosis only after predefined velocity thresholds are fulfilled. However, because APG deterioration triggered expedited Duplex assessment, the observed interval should be interpreted cautiously and cannot be considered an unbiased estimate of diagnostic lead time. Earlier investigations similarly suggested that plethysmographic deterioration may indicate compromised reconstruction or declining perfusion before overt clinical failure [15,17,25,26,27].

The predictive performance of serial changes in pulse volume amplitude deserves particular attention. A reduction in pulse volume amplitude of at least 25% from the one-month postoperative examination demonstrated good discrimination for subsequent fulfillment of the predefined Duplex criteria for significant restenosis. The one-month examination was selected as the postoperative reference to minimize the influence of acute perioperative changes while providing an early standardized assessment of the hemodynamic result of revascularization. Furthermore, postoperative APG deterioration remained the strongest independent predictor of procedural failure after adjustment for diabetes mellitus, lesion characteristics, treatment modality, and other clinically relevant variables. Although the proposed threshold requires external validation, including assessment across different anatomical and procedural subgroups, before widespread clinical adoption, these findings suggest that serial APG assessment may provide an objective physiological trigger for intensified surveillance or earlier diagnostic investigation.

The comparison between open surgical and endovascular treatment provides additional insight into postoperative hemodynamics. Both treatment strategies resulted in substantial early improvement in APG parameters, indicating that restoration of inline flow was associated with physiological improvement irrespective of the revascularization technique. However, patients undergoing open reconstruction demonstrated more sustained waveform stability during long-term follow-up, whereas the endovascular cohort exhibited a gradual decline that became more pronounced after approximately one year. Endovascular treatment was also independently associated with procedural failure in the multivariable analysis. This association should be interpreted cautiously, however, because treatment allocation was not randomized and residual confounding by anatomical complexity, lesion severity, or other factors influencing treatment selection may persist despite multivariable adjustment. Accordingly, the present findings should not be interpreted as demonstrating a causal disadvantage of endovascular treatment.

An additional advantage of APG lies in its relative independence from arterial compressibility. Because APG records volume displacement rather than arterial compression, it is considerably less affected by vessel incompressibility and therefore offers a complementary assessment in precisely those patient populations in whom conventional pressure measurements, like ABI, are least reliable [6,7,8,9,10,18]. Previous studies have demonstrated improved diagnostic accuracy when PVR is interpreted alongside ABI rather than in isolation, and our findings support this complementary approach during postoperative surveillance [6,28].

The results of this study should not be interpreted as suggesting that APG can replace Duplex ultrasonography. Duplex remains the reference standard for identifying the anatomical location, severity, and mechanism of restenosis and continues to guide therapeutic decision-making. Instead, APG should be viewed as an additional physiological surveillance tool that may identify patients requiring further investigation [1,5]. These findings raise the possibility that serial APG could be incorporated alongside routine clinical assessment to identify patients who may warrant closer Duplex surveillance. However, the present observational study was not designed to evaluate an APG-guided surveillance strategy, and whether such an approach could reduce imaging requirements, improve resource utilization, facilitate more appropriate reintervention, or improve clinical outcomes requires prospective evaluation.

The present study has several limitations. First, it was conducted at a single tertiary referral center, which may limit generalizability. Second, the relatively modest sample size and heterogeneous population, encompassing IC and CLTI, different anatomical disease patterns, and several open and endovascular procedures, limited meaningful subgroup analyses. Accordingly, the proposed ≥25% amplitude-reduction threshold should not be assumed to perform equivalently across clinical, anatomical, or procedural subgroups. Third, despite standardized acquisition, APG remains susceptible to environmental conditions and patient movement. In addition, APG deterioration triggered expedited Duplex assessment, resulting in differential surveillance intensity and potential ascertainment bias; consequently, the observed interval between APG deterioration and fulfillment of Duplex restenosis criteria should not be interpreted as an unbiased estimate of diagnostic lead time. Treatment allocation was non-randomized, and residual confounding by indication and anatomical complexity may have influenced comparisons between open and endovascular treatment. The conventional patient-level ROC analysis did not account for variation in event timing, while the limited number of procedural-failure events relative to the number of Cox-model covariates increases the possibility of overfitting. These diagnostic and multivariable findings should therefore be considered exploratory. Finally, although prespecified, the ≥25% amplitude-reduction threshold has not undergone external validation and requires confirmation in larger independent cohorts.

Despite these limitations, this study possesses several important strengths. To our knowledge, it represents the first contemporary prospective investigation comparing APG after both open and endovascular lower-extremity revascularization using a standardized surveillance protocol extending to 24 months. All patients underwent identical clinical, plethysmographic, ABI, and Duplex assessments at predefined intervals, allowing longitudinal evaluation of physiological recovery and direct comparison with conventional surveillance modalities. The integration of repeated-measures analysis, ROC analysis, survival analysis, and multivariable Cox regression further strengthens the robustness of our findings.

Future research should prioritize multicenter validation of APG-derived thresholds across different anatomical levels and revascularization techniques, alongside development of automated digital waveform analysis and artificial-intelligence approaches capable of recognizing subtle waveform changes. Combining APG with Duplex ultrasonography, toe pressure measurements, transcutaneous oxygen tension, or near-infrared spectroscopy may ultimately provide a comprehensive physiological assessment of both macrovascular and microvascular recovery after lower-extremity revascularization. External validation of the proposed 25% threshold, including evaluation across different anatomical levels and revascularization techniques, should represent a priority of future multicenter investigations. As Vascular Surgery increasingly emphasizes objective functional outcomes in addition to anatomical success, APG may once again assume a meaningful role within modern postoperative surveillance pathways.

## 5. Conclusions

APG provides a reproducible and clinically meaningful evaluation of lower-extremity hemodynamics following peripheral arterial revascularization. In this prospective study, APG demonstrated significant functional improvement after both open surgical and endovascular interventions, correlated with conventional hemodynamic parameters, and identified physiological deterioration that frequently preceded fulfillment of the predefined Duplex criteria for significant restenosis. Postoperative APG deterioration was also independently associated with procedural failure and reduced long-term primary patency. These findings suggest that APG should not be regarded as a replacement for Duplex ultrasonography, but rather as a potentially complementary physiological surveillance modality capable of identifying hemodynamic deterioration that may warrant further diagnostic evaluation. Given its non-invasive nature, reproducibility, relative independence from arterial calcification, and ability to provide objective functional information, APG may represent a useful complementary tool within contemporary postoperative surveillance pathways. Larger multicenter prospective studies are warranted to validate these findings, establish standardized diagnostic thresholds, and define the precise role of APG within contemporary vascular practice.

## Figures and Tables

**Table 1:** Baseline demographic, clinical, hemodynamic, and lesion characteristics of the study population according to treatment strategy.

Variable	Overall (*n* = 120)	Open (*n* = 60)	Endovascular (*n* = 60)	*p*-Value
Age, years	69.1 ± 8.7	69.8 ± 8.4	68.4 ± 9.0	0.384
Male sex	84 (70.0%)	43 (71.7%)	41 (68.3%)	0.689
Body mass index, kg/m^2^	28.3 ± 4.5	28.6 ± 4.4	28.0 ± 4.7	0.476
Current/former smoker	76 (63.3%)	39 (65.0%)	37 (61.7%)	0.709
Diabetes mellitus	55 (45.8%)	30 (50.0%)	25 (41.7%)	0.360
Hypertension	95 (79.2%)	49 (81.7%)	46 (76.7%)	0.501
Dyslipidemia	87 (72.5%)	45 (75.0%)	42 (70.0%)	0.542
Coronary artery disease	42 (35.0%)	23 (38.3%)	19 (31.7%)	0.442
Chronic kidney disease	23 (19.2%)	13 (21.7%)	10 (16.7%)	0.486
Previous ipsilateral intervention	21 (17.5%)	9 (15.0%)	12 (20.0%)	0.473
Intermittent claudication	47 (39.2%)	24 (40.0%)	23 (38.3%)	0.852
Chronic limb-threatening ischemia	73 (60.8%)	36 (60.0%)	37 (61.7%)	0.852
Baseline ABI	0.54 ± 0.16	0.53 ± 0.15	0.55 ± 0.17	0.420
Target lesion length, cm	14.2 ± 6.8	14.9 ± 7.1	13.5 ± 6.4	0.274
Chronic total occlusion	58 (48.3%)	31 (51.7%)	27 (45.0%)	0.465
Femoropopliteal disease	68 (56.7%)	33 (55.0%)	35 (58.3%)	0.714
Isolated infrapopliteal disease	18 (15.0%)	10 (16.7%)	8 (13.3%)	0.603
Multilevel disease	34 (28.3%)	17 (28.3%)	17 (28.3%)	1.000
Patent runoff vessels	2.1 ± 0.8	2.0 ± 0.8	2.2 ± 0.8	0.212

Categorical variables are presented as *n* (%), while continuous variables are presented as mean ± STD. Abbreviation: ABI: ankle-brachial index.

**Table 2:** Multivariable Cox proportional hazards regression analysis of predictors of procedural failure.

Variable	Exp(B)	95% CI	*p*-Value
APG deterioration ≥25%	2.80	1.65–5.37	**<0.001**
Diabetes mellitus	2.14	1.16–3.93	**0.015**
Endovascular treatment	1.81	1.04–3.17	**0.037**
Chronic kidney disease	1.58	0.84–2.98	0.160
Current or former smoking	1.39	0.75–2.58	0.300
Lesion length, per 5 cm increase	1.18	0.93–1.50	0.170
Baseline ABI, per 0.1-unit increase	0.92	0.74–1.15	0.470

Statistically significant *p*-values appear in bold. Abbreviations: ABI: ankle-brachial index; APG: air plethysmography.

## Data Availability

Data is available by the corresponding author.

## Abbreviations

The following abbreviations are used in this manuscript:

ABI	Ankle–brachial index
APG	Air plethysmography
CKD	Chronic kidney disease
CI	Confidence interval
CLTI	Chronic limb-threatening ischemia
CTA	Computed tomography angiography
HR	Hazard ratio
ICC	Intraclass correlation coefficient
IC	Intermittent claudication
PAD	Peripheral arterial disease
PSV	Peak systolic velocity
PVR	Pulse volume recording
ROC	Receiver operating characteristic
STROBE	Strengthening the Reporting of Observational Studies in Epidemiology
TLR	Target lesion revascularization
